# Neural Correlates of Morphology Acquisition through a Statistical Learning Paradigm

**DOI:** 10.3389/fpsyg.2017.01234

**Published:** 2017-07-27

**Authors:** Michelle Sandoval, Dianne Patterson, Huanping Dai, Christopher J. Vance, Elena Plante

**Affiliations:** Department of Speech, Language, and Hearing Sciences, University of Arizona, Tucson AZ, United States

**Keywords:** statistical learning, implicit learning, language, morphology, fMRI, second language acquisition

## Abstract

The neural basis of statistical learning as it occurs over time was explored with stimuli drawn from a natural language (Russian nouns). The input reflected the “rules” for marking categories of gendered nouns, without making participants explicitly aware of the nature of what they were to learn. Participants were scanned while listening to a series of gender-marked nouns during four sequential scans, and were tested for their learning immediately after each scan. Although participants were not told the nature of the learning task, they exhibited learning after their initial exposure to the stimuli. Independent component analysis of the brain data revealed five task-related sub-networks. Unlike prior statistical learning studies of word segmentation, this morphological learning task robustly activated the inferior frontal gyrus during the learning period. This region was represented in multiple independent components, suggesting it functions as a network hub for this type of learning. Moreover, the results suggest that subnetworks activated by statistical learning are driven by the nature of the input, rather than reflecting a general statistical learning system.

## Introduction

The discovery that individuals can learn about the structure of a language by tracking distributional information in the input fueled new inquiries into the nature of language acquisition in children and in adults. Learning from distributional information in language input is typically considered a type of statistical learning (see Karmiloff-Smith et al., 1998; Saffran, 2003; Erickson and Theissen, 2015 for overviews). Behavioral evidence supports a role for statistical learning in many aspects of language acquisition including phonology (e.g., Maye et al., 2002), word learning (e.g., Saffran et al., 1996; Saffran, 2001; Smith and Yu, 2008) and morpho-syntactic development (e.g., Gerken et al., 2005; Thompson and Newport, 2007; Christensen et al., 2012).

Electrophysiological studies have confirmed that rapid statistical learning of word units is accompanied by equally, if not more rapid physiologic changes in brain function. Cunillera et al. (2009) showed physiologic evidence of implicit word segmentation from running speech within a minute of exposure to non-word strings. Likewise, physiological evidence of learning occurs after minutes of exposure to words containing morpheme-like features (De Diego Balaguer et al., 2007; Mueller et al., 2009; Havas et al., 2017). Others (McLaughlin et al., 2004) have shown that physiologic change can precede behavioral change, representing a neural precursor for learning.

Functional imaging studies have begun to uncover spatially defined networks associated with distributional learning. To date, learning paradigms have been limited to discovery of words forms (McNealy et al., 2006; Cunillera et al., 2009; McNealy et al., 2010; McNealy et al., 2011; Karuza et al., 2013; Plante et al., 2015). Specifically, these studies have identified networks involved in segmenting word units from running speech, which typically lacks clear acoustic cues to word boundaries. However, neuroimaging inquiry into statistical learning has been slow to move beyond word segmentation, which is thought to reflect the earliest phase of natural language learning.

There have been several imaging studies involving artificial syntactic rules (e.g., Forkstam et al., 2006; Newman-Norlund et al., 2006; Bahlman et al., 2008; Hauser et al., 2012; Petersson et al., 2012; Folia and Petersson, 2014). However, these largely employed training outside the scanner, and scans were acquired during a test phase rather than the learning phase. Therefore, learning and testing effects cannot easily be dissociated, a concern given test-specific effects have been documented elsewhere (e.g., Thiel et al., 2003; Petersson et al., 2004). One syntax-learning study did scan participants during the learning phase, presenting sentences to learners in printed format. In this study, participants were exposed to both grammatical and ungrammatical word combinations during the same scans, and these two conditions were compared (Tettamanti et al., 2002). They reported an inferior frontal-prefrontal-supramarginal/angular network for grammatical learning. We note that the presence of both types of word combinations can alter learning through interference from incorrect exemplars (Gómez and Lakusta, 2004; Criss et al., 2011), raising a question concerning how typical these findings might be for learning of correct grammatical forms only. Interestingly, however, activation increased in the inferior frontal gyrus (pars opercularis) during learning of grammatical rules in the Tettamanti et al. (2002) study, suggesting correspondence to increased engagement in learning over time. Here we focus on implicit learning only through exposure to correct syntactic categories that are marked by morphological suffixes. We used natural language stimuli that represent a subset of the Russian gender marking system. Functional magnetic resonance imaging (fMRI) was used to reveal the neural correlates of distributional learning related to this level of linguistic representation.

### Statistical Learning of Morphologically-Defined Linguistic Categories

Languages divide words into discrete classes referred to as syntactic categories (e.g., nouns, verbs, and adjectives). Our ability to combine words into novel sentences is dependent on the acquisition of these categories. Without them, the combinatorial rules of language would operate on a word-by-word basis, making linguistic generativity impossible. The categories of noun and verb are common across languages, but languages may also include word subcategories. For example in many languages (e.g., Russian, Norwegian, French, and Spanish, but not English), finer distinctions are made between nouns of different gender categories (e.g., feminine, masculine, and neutral). Subcategories, as learners of foreign languages can attest, can be particularly difficult to master. Grammatical gender subcategories, in particular, are often replete with semantic inconsistencies (Bock, 1982). Although the gender subcategory of specific words is arbitrary (e.g., *book* is masculine in Spanish – *el libro* but feminine in Russian – *kniga*), gender marking within a language is often signaled by one or more morphological or phonological markers. For instance in Spanish, masculine nouns often carry one or more markers such as *el* and –*o*, but they do not always refer to overtly masculine concepts (e.g., book). For this reason it has been suggested that distributional learning—tracking distributional patterns such as morphological, phonological, and positional regularities—may be instrumental in the acquisition of gender classes (e.g., Karmiloff-Smith, 1979) as well as broader word categories like nouns and verbs (Mintz, 2003).

Behavioral work on distribution-based category formation has revealed that in order for learners to jump from tracking surface associations (e.g., in Spanish: el gato; la fruta) to inferring word categories (e.g., masculine, feminine). The neurophysiological reality of this dual learning is reflected by different ERP components that emerge in relation to word identity and word morphology (De Diego Balaguer et al., 2007; Mueller et al., 2009; Havas et al., 2017). To accomplish this, learners require converging or overlapping distributional regularities (Braine, 1987; Frigo and McDonald, 1998; Gerken et al., 2005). When artificial languages are created to abide by these cognitive constraints, infants and adults form categories quickly and implicitly. Moreover, learners can use their knowledge of category properties to generalize to new words (Mintz, 2002; Gómez and Lakusta, 2004; Gerken et al., 2005; Richardson et al., 2006; Eidsvåg et al., 2015). These redundant, overlapping regularities are present in natural languages and have been shown to facilitate the acquisition of language categories by non-native speakers (Richardson et al., 2006; Eidsvåg et al., 2015). Like distributional-based word segmentation (Saffran et al., 1996), the distributional learning of word classes occurs quickly, without feedback, and the underlying mechanism operates in both infancy and adulthood (Mintz, 2002; Gómez and Lakusta, 2004; Gerken et al., 2005; Richardson et al., 2006).

Russian provides a natural language context in which overlapping and redundant cues are available to different degrees for gender subcategories (see **Table 1**). In Russian, nouns can take one or the other of two suffixes that mark the noun’s gender (e.g., masculine nouns can end in *–ya* or –*yem*; feminine nouns can end in –*oj* or -*u*). Therefore, the distribution of these pairs of endings provides converging evidence for a noun’s subcategory as either masculine or feminine. In addition, some nouns may be double marked for gender by additional gendered suffixes (e.g., -*tel* ++*yem* may mark a masculine noun and -*k* +*oj* a feminine noun). Double-marked nouns offer a higher degree of overlapping and redundant cues to gender subcategory membership than do the single-marked nouns.

**Table 1:** Example words from the Russian stimulus set.

	Double-marked	Single-marked
Masculine (+tel, +ya/yem)	vodi + **tel + ya**	fonar + **-ya**
	vodi + **tel + yem**	fonar + **-yem**
Feminine (+k, +oj/u)	blondin + **k + oj**	zabav + **-oj**
	blondin + **k +u**	zabav + **u**

When exposed to a set of Russian nouns both English-speaking adult and English-learning infants discriminated between grammatical and ungrammatical examples of the Russian gender system (Gerken et al., 2005; Richardson et al., 2006; Eidsvåg et al., 2015). A portion of the noun-inflection combinations were withheld from training and included at test to measure generalization to novel words. Participants generalized suggesting that discrimination was not solely due to the learner’s familiarity with the words. Instead, generalization indicated the formation of a subcategory. Although the Russian gender marking system can be more difficult to learn relative to other gender systems (e.g., Norwegian; Rodina and Westergaard, 2017), Gerken et al. (2005) described the presence of redundant “double-marked” words as especially facilitative of learning and generalization. Gerken et al. (2005) found no such generalization in conditions in which infants were provided with only single marked nouns during the learning phase. In addition several studies of adult learners have shown better performance for double marked vs. single marked words (Gerken et al., 2005; Richardson et al., 2006; Eidsvåg et al., 2015).

The word category paradigms involve tracking co-occurrence associations within words (e.g., noun+suffix and noun+suffix+suffix), but they also require tracking the similarity of these associations across words (e.g., words and suffixes that take –*ya* can also take –*yem*, see **Table 1** for examples). Therefore, this task requires tracking of adjacent contingencies, but in addition requires tracking of relations among elements (e.g., -*ya* and *–yem* are both associated with the word *fonar*, but not *zabav*; both *–u* and *–oj* can follow *–k*, when it appears, but not *–tel*). Given that this type of tracking is conceptually different than that required for discovering the syllable co-occurrences that define words, it is an open question whether the brain would recruit the same network for both types of distributional processes.

### Neuroimaging Studies of Statistical Learning

Neuroimaging studies (fMRI) of statistical learning have primarily examined word segmentation (McNealy et al., 2006, 2010; Cunillera et al., 2009; Karuza et al., 2013; López-Barroso et al., 2015; Plante et al., 2015). In these studies, the stimuli are created to facilitate learning via transitional probabilities. Regardless of whether natural or artificial language stimuli are used, we see consistent activation in the superior temporal gyrus during learning (McNealy et al., 2006, 2010; Cunillera et al., 2009; Karuza et al., 2013; López-Barroso et al., 2015; Plante et al., 2015). The activation appears to be linked to distributional learning and not just general auditory processing. Indeed, activation in this region is stronger both when compared to non-linguistic auditory input and when comparing conditions in which contain syllable-level dependencies are present or absent (Cunillera et al., 2009; Plante et al., 2015).

The left inferior frontal gyrus, including Broca’s area, has also been argued to be important for learning via transitional probabilities when stimuli are auditory-linguistic (Karuza et al., 2013) or visual (Turk-Browne et al., 2009). However, activation in this area and others (e.g., frontal regions, and areas in the temporo-parietal-occipital junction) is not consistently evident across or within word segmentation studies (Plante et al., 2015). For example, two studies (McNealy et al., 2006; Karuza et al., 2013) reported activation in this area not during exposure, but in relation to performance on a post-scan test. Plante et al. (2015) reported inferior frontal activation, which was weaker and less consistently present than temporal lobe activation. They suggested inferior frontal activation could be related to periodic violations of expectancies that occur as patterns are acquired (cf. Petersson et al., 2004). This explanation would also potentially explain the Karuza et al. (2013) findings as well as lack of frontal findings in other word segmentation studies. However, activation in Broca’s area is frequently reported in artificial syntax studies, including the one study to examine morphosyntactic learning during scanning (Tettamanti et al., 2002). These paradoxical findings raise the issue of whether activation of this region is specific to certain types of distributional information in the input.

We hypothesize stronger contributions of inferior frontal activation for learning bound morphemes than was seen previously for word segmentation learning paradigms. Previous fMRI studies have argued that the left inferior frontal cortex (i.e., the pars opercularis and the pars triangularis) is critical for the morphological parsing (e.g., Tyler et al., 2004; Joanisse and Seidenberg, 2005; Slioussar et al., 2014). Patients with left inferior frontal cortex damage show impairment on tasks involving verb tense morphology [see Faroqi-Shah (2007) meta-analysis] as well as for case and gender errors (Bates et al., 1987). This indicates a central role for Broca’s area in the processing of morphological units in general. This region may also be central during learning of an inflectional morpheme system for gender categories. It is important to note that Broca’s area also implicated in processing or use of irregular verbs (Joanisse and Seidenberg, 2005; Faroqi-Shah, 2007). From a statistical learning perspective, these verbs may be learned as associations between the root verb and it’s irregular pair. If this is the case, the inferior frontal gyrus may serve multiple aspects of language through a common statistical learning function.

### The Present Study

In the present study we use functional magnetic resonance imaging (MRI) during learning of a gender system, to uncover the neural correlates of word class acquisition via statistical learning. To prevent learners from using their pre-existing language knowledge, this study examines adults as they learn the gender system of an unfamiliar language (Russian) and an unfamiliar word class distinction (gender, a distinction English does not employ). We examine adults rather than children to dissociate acquisition processes from maturational ones. We predict that activation in the superior temporal gyrus, documented for all auditory statistical learning studies to date will also characterize learning of gender subcategories. However, we expect to find novel patterns of activation associated with learning an inflectional morphology system. Given the particular role of morphological endings for identifying subcategory membership, we predict that the brain will recruit areas of the left inferior frontal cortex, particularly pars opercularis (Tettamanti et al., 2002; Goucha and Friederici, 2015).

## Materials and Methods

### Participants

Eighteen subjects participated in the present study (*M*_age_ = 24 years; range = 18–38 years). Thirteen participants were female and five were male. Three participants were left-handed (two females and one male). Two of these had left lateralized language on the experimental task and one had largely symmetrical language representation. However, all three were retained in the study so that the subject sample would resemble the full population of interest (adults, regardless of handedness). Participants were screened for abnormal hearing, a history of language difficulties, head injury, psychiatric medications and MR safety concerns before being permitted to participate. We excluded one potential participant who had movement in all four scans, averaging greater than 1 mm (1.6–9 mm), who was replaced in the final dataset. The research is approved by the University of Arizona Institutional Review Board and all participants provided informed consent.

### Materials

#### Tone Stimuli

Complex tones were used as an auditory control condition for Russian words. Tones reflected the range of durations of the word stimuli *M* = 655 ms, range 500–880 ms). Each tone was developed by starting with pairs of pure tones of equal duration. One pure tone of the pair fell in the range of 500–800 Hz and the other from 1000 to 3000 Hz to reflect frequencies in the speech range. Tones were edited to provide approximately equal loudness, despite their frequency differences. Each pure tone was then frequency and amplitude modulated and the paired tones were blended. Twenty complex tones were presented within a tone block, with interstimulus intervals designed to mimic that used in the word blocks (described below).

#### Learning Stimuli

One hundred and sixty multi-syllable Russian words were recoded by one female and one male native Russian talker. These words were divided equally between masculine and feminine nouns and words taking single and double markings. **Table 2** presents examples of these stimuli. Sound files were computer-edited to minimize background noise in the recordings and to produce approximately equal loudness across word tokens. Twenty unique words were heard per stimulus block. Each root word was heard with its two alternate gender suffixes (i.e., single marked: -u, -oj, -ya –yem; double marked: -ku, -koj, -telya, -telyem) and spoken by the same talker (i.e., male or female) within the same stimulus block. Inter-stimulus intervals were jittered so that 20 spoken words of different durations fit within an 18 s block.

**Table 2:** Examples of the training and test words.

Stimulus type	Masculine	Feminine
In-Scanner Exposure phase		
Ending 1: Double marked	dushi**telya**	karmel**koj**
Single marked	kon**ya**	gazeta**oj**
Ending 2: Double marked	dushi**telyem**	karmel**ku**
Single marked	kon**yem**	gazeta**u**
Pre-Test Familiarization		
Familiarization	pokupa**telya**	devush**koj**
	uroven**yem**	voron**u**
Prescan and Post-Scan Test stimuli		
Correct	pokupa**telyem**	devush**ku**
	uroven**ya**	voron**oj**
Incorrect	pokupa**telu**	devush**kyem**
	uroven**oj**	voron**ya**

Stimuli heard during scanning consisted of eight blocks of tones interleaved with eight blocks of spoken Russian. Tone stimuli preceded word stimuli so that participants had an opportunity to settle into the scan prior to the onset of learning. Each block consisted of a 4 s period of silence followed by a brief instructional cue, 18 s tone input, another 4 s of silence and instructional cue, and 18 s of Russian word input. The instructional cue for the tone block was “Just relax” and the instructional cue for the word blocks was “Listen to these words.” Participants received a total of 2.4 min of exposure to Russian words during each scan.

#### Post-scan Familiarization Stimuli

A second female talker recorded 24 Russian root+gendered suffix pairs. These words were not included in the Exposure stimulus set. Root words were recorded with only one of their correct gendered suffixes, with half the words reflecting masculine/feminine and single/double marked forms. Words were computer edited to approximate equal loudness and to reduce background noise. Words were arranged into an audio file with each word heard twice, but never consecutively. See **Table 2** for examples.

#### Test Stimuli

Twenty-four stimuli consisted of the root words presented during post-scan familiarization but with alternate suffixes. These were recorded by the same female talker who recorded the post-scan familiarization stimuli, and stimuli were computer edited as described previously. Half of the root words were recorded both with their correct gender markings (e.g., masculine word + masculine suffix). For these, the gender markings used were the alternate marking for that provided in the post-scan familiarization stimuli (e.g., post-scan familiarization: korenya; test: korenyem). Half of the words were incorrect in that root word was paired with correct number of suffixes (i.e., single-marked, double-marked), but the suffix heard was the incorrect gender (masculine vs. feminine). These words and their gendered markings were never heard as part of the learning stimuli, and only the root words with one of the two possible gender markings were heard during as part of the post-scan familiarization stimuli. Thus, the test words prompted generalization of the patterns encountered during the scans, rather than memory for specific words heard previously. These stimuli were used in both a pretest and for post-scan testing. See **Table 2** for examples.

#### Training Stimuli

The female native Russian talker who recorded the post-scan exposure and test items also recorded a series of verbs and adjectives used during prescan training. This voice was not heard during learning. The prescan stimuli provided input during training that was phonetically similar to the training phase, but did not provide the gender information targeted for learning. These were used to familiarize each participant with the experimental procedures prior to scanning.

### Behavioral Procedures

The experiment consisted of five phases: (1) A prescan training phase was used to acquaint participants with the experimental procedures, (2) A pretest of the Russian gender system to provide baseline response rates prior to scanning, (3) A learning phase during which participants were exposed to a set of Russian words and simultaneously scanned, (4) a post-scan exposure to additional Russian root words, and (5) The test phase used to assess the degree of learning after the exposure scan. Phases 3–5 were repeated four times during the experimental session.

#### Prescan Training

The experimental procedures were explained to the participants, including that they would be tested on what they learned. They were not provided any further information concerning the nature of the words they would be hearing, what they should attend to in the stimuli, or what aspect of the stimuli they would be tested on. Participants then listened to the training stimuli so that they were familiar with the format of the experiment.

#### Pretest

A pretest was administered to establish the base rate of response (the guessing rate) to the test items used after each scan. Participants listened to the 24 test stimuli presented in computer-randomized order. Participants indicated, via key press on a computer keyboard, whether the test item was correct of incorrect. Responses were recorded using DirectRT (Jarvis, 2012). No feedback was provided.

#### Learning Phase

Participants heard tone and Russian stimuli during a scan designed to assess learning networks. The onset of each scan was triggered by a scanner signal to provide precise timing control. Before the scan, participants were again instructed to listen during scanning and that no response was required during this part of the study, buy they would be tested on what they had learned after each scan. Scans were repeated four times during the experimental session.

#### Post-scan familiarization

Immediately after each scan, and while still in the scanner, participants listened to the post-scan familiarization audio-file. They were told to listen to these words and that these were the words they would be tested on. No scan data was acquired during this time.

#### Post-scan Testing

Testing occurred immediately after the post-scan familiarization phase and while the participant was still in the scanner. No scan data was acquired during this time. Participants were given the same two-alternative forced choice test that was given in the pretest phase (see above).

### Imaging Procedures

Participants were scanned on a 3 tesla Siemens Skyra using a 32-channel head coil and running on software version syngo MR D13. Participants heard the stimuli via noise attenuating MR compatible Sensimetrics S14 Insert Earphones for fMRI Research. Each of the four fMRI scans lasted 6 min and 4 s. The total time for the experiment was about 1 h, with time for a break in the middle if the participant was uncomfortable.

#### Image Acquisition Parameters

Four fMRI scans were collected in axial orientation using an interleaved inferior to superior echo-planar free induction decay sequence and GRAPPA2 (TR 2000, TE 30, Flip angle 90, FOV 241.92 × 201.6, Matrix 72 mm × 60 mm × 36 mm, voxel size 3.36 × 3.36 × 4). Each scan was 182 volumes (including 6 pre-stimulus volumes). To aid in boundary-based registration (Greve and Fischl, 2009) of both fMRI and DWI sequences, we collected a fieldmap with one phase and two magnitude images (TR 434, TE 4.92 [first magnitude image], 7.38 [second magnitude image and phase image], Flip angle 60, FOV 224, Matrix 68 × 68 × 41). A high resolution MPRAGE was collected left to right in the sagittal plane using GRAPPA2 (TR 2300, TE 2.95, Flip angle 9, FOV 176 × 240, Matrix 176 × 240 × 256). This was critical for registration into standard space.

#### fMRI Preprocessing

We preprocessed fMRI data in AFNI (Version AFNI_16.0.00, Cox, 2012) by first trimming the first six volumes from the scan, which were included to ensure initial patient and scanner stability prior to the onset of data collection. Realignment of the volumes in the scan was accomplished with SLOMOCO (Beall and Lowe, 2014). SLOMOCO combines within-volume slicewise correction with 3D volume registration. Because realignment can only correct for small movements and rotations (Cox and Jesmanowicz, 1999), we modified the SLOMOCO algorithm by optimizing the reference volume. Using information from AFNI, we selected a highly stable and representative scan volume to be used for this purpose in SLOMOCO. This slice selection step halved the amount of movement required for realignment of each target volume. After realignment we applied despiking in AFNI. We then used boundary based registration in FSL (Jenkinson et al., 2012) to register each participant’s scans to their MPRAGE and then into standard space. Boundary based registration uses the gray–white matter boundaries for registration as these tend to be more reliable than the external gray matter boundaries (Greve and Fischl, 2009). Finally we smoothed the standard space image to 6 mm.

#### fMRI Analysis

Independent component analysis (ICA) separates the raw BOLD signal obtained across the full scan time period into a set of statistically independent periodic signal sources. Beta weights are then calculated between the ICA models representing independent signal sources and signal from the actual data. ICA is model free in that the it does not rely on an *a priori* model of the BOLD signal, but derives signal time courses and magnitude from the data. We used GIFT (Version 4.0a; The Gift Documentation Team, 2015), to run the Entropy Rate-Based Minimization (ERBM) ICA algorithm because it performs Joint Blind Source Separation. JBSS improves the back reconstruction of data as compared to Infomax (Li and Adali, 2010) enabling calculation of subject-by-subject ICA data via back reconstruction. We were especially interested in the back reconstruction because we anticipated activation differences from scan to scan as participants learned. We ran ERBM with 50 ICs to help provide refined components that better correspond to known structural and functional divisions (Allen et al., 2014). Signal change was represented in *z*-scores. After running ERBM we ran the Icasso program, using 10 iterations of the ICA analysis. Icasso then provides metrics of component stability and statistical overlap.

Because ICA identifies periodic signals within the BOLD response, regardless of origin, it was necessary to separated task-related ICs from artifact. This involved several convergent methods. We sorted the ICs by the degree to which their beta weights indicated significantly greater signal during the word stimuli relative to the tone stimuli. We retained ICs for which the Beta weight reflecting the association between the task period and the BOLD signal were statistically non-zero (one-sample *t*-test, *p* < 0.05, uncorrected for multiple comparisons) in at least one of the four scans. This allowed for the possibility that some areas might be needed either early or late in learning, but not throughout learning. This criterion reduced candidate ICs from 50 to 32.

We further assessed the stability, the replicability, and the statistical overlap among the remaining components. One of the remaining ICs failed to replicate across the ten iterations of the ICA procedure. We used the Icasso component similarity graph to insure that we chose only components that were compact and isolated across all 10 iterations Icasso (i.e., no overlap with other components). This assured true statistical independence of the components. Three additional components were eliminated on this basis. We used the Icasso stability estimate (Iq) in GIFT to identify and reject components with an Iq < 0.80. This eliminated two additional components. Visual inspection of these two components indicated that the greatest signal in these ICs were associated with pulsatility in the basal ventricular system or blood vessel pulsatility at the level of the pons. We also inspected the power spectra of the remaining ICs to insure that the components we chose had good dynamic range and power ratio (Allen et al., 2011). Finally, we visually inspected spatial activation maps to identify ICs that had activation primarily in white matter or CSF, and ICs that were characterized by susceptibility artifact or rim activation at the cortical edge associated with movement. These were eliminated from further consideration. This process left five ICs available for further analysis.

## Results

### Behavioral Results

**FIGURE 1:**
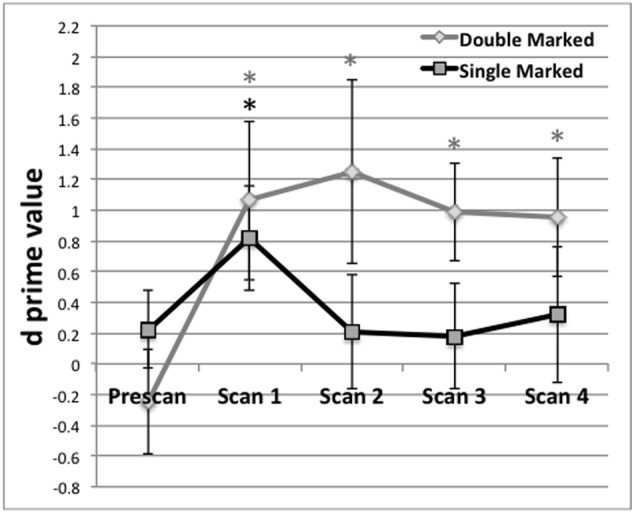
Behavioral performance (*d* prime) on test blocks administered before the first scan (Prescan) and after scans 1–4. Mean behavioral performance for Double (black) and Single Marked (gray) words. A black asterisk indicates recognition of Single Marked words was above chance performance. A gray asterisk indicates the same for Double Marked words. Error bars reflect the SEM.

The *d*-prime scores for double- and single-marked words were calculated for each participant, for each test. The *d*-prime data are displayed in **Figure 1**. Results for the Pretest indicated that responses for the double-marked items did not differ from chance, *t*(1,17) = -0.77, *p* = 0.2252, nor did results from the single-marked items, *t*(1,17) = 0.94, *p* = 0.1752. As **Figure 1** indicates, performance for each item type overlapped and straddled the zero (or chance) line at pretest, indicating that participants were not able to correctly guess test items. Responses to double- and single-marked words also did not differ from each other, *t*(1,17) = 1.72, *p* = 0.2568.

For each post-scan behavioral test, we evaluated the *d′* values to determine whether they exceeded chance performance. Post-scan test 1, obtained after the first scan, exceeded chance for both double- *t*(1,17) = 2.16, *p* = 0.0228) and single-marked words *t*(1,17) = 2.46, *p* = 0.0130). Performance on double-marked words remained above chance for the remainder of the experiment (*p* < 0.02 in all cases). However, performance on single-marked test items fell below chance after Scan 1 and remained there for the remaining scans (*p* > 0.45 in all cases).

Differences on double- and single-marked test items across time were assessed using a repeated-measures ANOVA with Marker Type (Double vs. Single) and Time (Scans 1–4) as within-subject conditions. We found a main effect of Marking Type, *F*(1,15) = 14.79, *p* = 0.0076, with average performance on double-marked words (*M* = 1.02, *SE* = 0.24) exceeding average performance on single-marked words (*M* = 0.39, *SE* = 0.25). The main effect Time did not reach significance, *F*(3,45) < 0.42, *p* = 0.7389 nor did the Marker by Time interaction, *F*(3,45) < 0.21, *p* = 0.8898.

### Imaging Results

**FIGURE 2:**
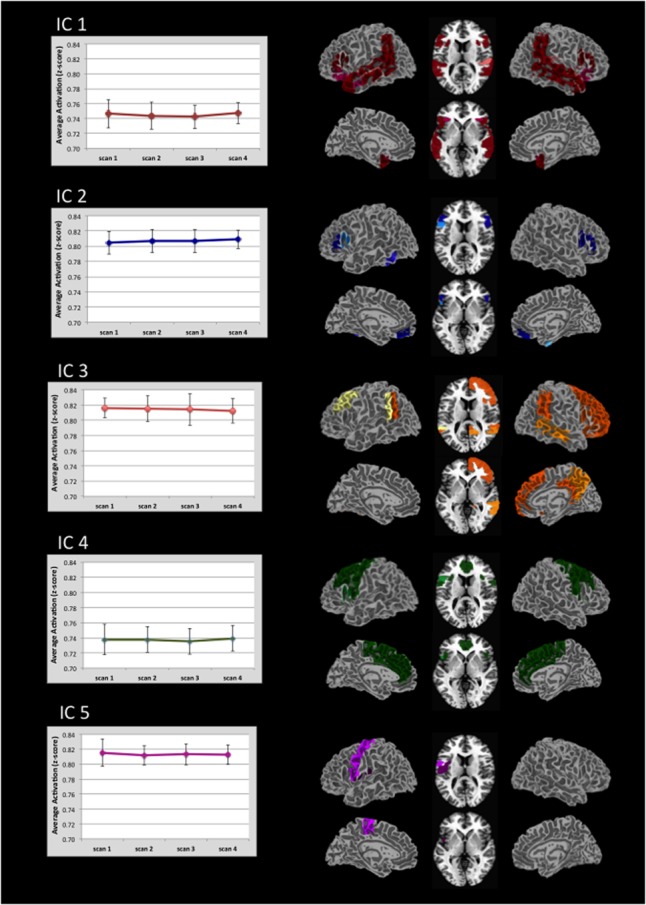
Regional distribution of activation in five task-related ICs and there associated level of activation. Activation for each IC is thresholded at *p* < 0.05 (FWE corrected). Darker colors indicate areas of greatest overlap of regional activation across the four scans (see also Supplementary Table 1). Graphs indicate the average level of activation across all voxels within the IC. Note that the value range is truncated in the graphs. Error bars indicate ±1 standard deviation.

**Figure 2** displays the alpha corrected (*p* < 0.05, FWE correction) activation patterns for each IC. As this figure suggests, ICs were remarkably stable in terms of the regions activated and their degree of activation across the four scans. Each image represents an IC with the color gradient (lighter to darker) representing increasing overlap of activation across the four scans. Supplementary Table 1 contains the maximum *t*-values and their *x, y, z* locations for every significantly active region broken down by IC and scan within predefined regions defined by the Harvard-Oxford atlas. We briefly summarize the major areas of activation for each IC here. Supplementary Figure 1 provides the spatial map of the results of a GLM analysis using the same data for comparison purposes. As expected, the GLM analysis returned much more limited activation than did the ICA.

For IC 1, the strongest activation occurred along the length of the planum temporale, superior temporal gyrus, posterior supramarginal and angular gyri, and the posterior middle temporal gyrus. This activation was represented bilaterally. Additional activation was distributed in the frontal, temporal, and parietal-occipital cortices.

IC 2 was characterized primarily by activation in the inferior frontal gyrus (pars opercularis and pars triangularis). Activation within pars opercularis was right lateralized whereas pars triangularis was nearly equal across hemispheres.

Activation in IC 3 included bilateral activation in the posterior superior temporal and angular gyri, as well as right hemisphere activation in multiple frontal regions (orbital frontal, frontal pole, superior and middle frontal gyri, paracingulate and posterior cingulate gyri), and right lateral occipital gyrus. Consistent activation in the left Crus I and Crus II was also seen.

IC 4 showed left hemisphere activation in inferior frontal gyrus-pars opercularis, central opercular cortex and precentral gyrus. Bilateral activation also occurred in the anterior cingulate, juxtapositional lobule, paracingulate, precentral, and superior frontal gyri.

IC 5 showed left hemisphere activation of the pre-and post-central gyri. Bilateral activation was also present in the central opercular cortex.

**FIGURE 3:**
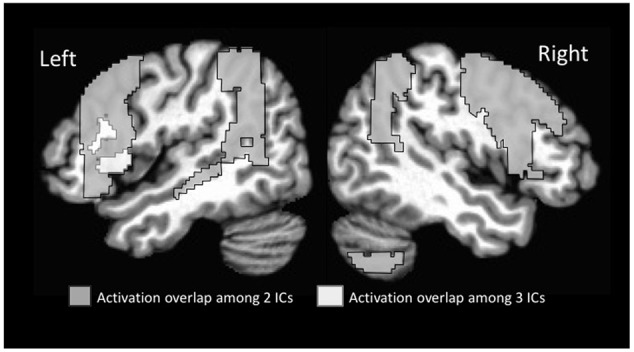
Overlap among regions activated on multiple time courses (ICs).

Of particular note relative to our hypothesis was that the inferior frontal gyrus was active across multiple ICs (see Supplementary Table 1 for activation strength in the different subregions of this gyrus). Given the particular role of morphological endings for identifying subcategory membership, we predicted that the left inferior frontal cortex would be recruited to a greater extent than previously seen in statistical learning fMRI studies that examined word segmentation. In the present study, the left inferior frontal gyrus-pars opercularis was active in four of the five ICs (1, 2, 4, 5). The right inferior frontal gyrus-pars opercularis was likewise active in four ICs (1, 2, 3, 4). All overlapping regions displayed in **Figure 3**. For the Inferior Frontal Gryus, the majority of overlapping portions encompassed the superior aspect of pars opercularis/pars triangularis as well as an inferior portion that extended into the anterior insula. This degree of overlap suggests that the inferior frontal gyrus may have served as a hub in the learning network for morphologically-defined gender subcategories.

Additional regions showed overlap among two different ICs. These included additional sub-regions of the inferior frontal gyrus (pars triangularis extending into pars orbitalis, and opercularis) in the left and pars opercularis in the right. In addition, regions in the middle frontal gyrus were active bilaterally along with the full extent of the temporo-parietal-occipital junction bilaterally. The latter region included the angular gyrus, posterior supramarginal gyrus, and superior lateral occipital gyrus.

### Activation and Post-scan Testing

To gain converging evidence for the centrality of the inferior frontal gyrus for Russian morphology learning, we correlated behavioral performance with activation level in the inferior frontal gyrus and other commonly activated regions (see **Figure 3**), for each IC and scan in which they occurred. We extracted activations that were statistically significant using masks of the inferior frontal sub-region derived from the Harvard-Oxford cortical structural atlas. The average activation (*t* statistic) within these regions was extracted on a subject-by-subject and scan-by-scan basis. These were correlated with individual subject performance on double- and single-marked test items using a Pearson product moment correlation (a total of 24 correlations for 3 inferior frontal regions × 2 learning measures × 4 scans). The reported correlations are significant at *p* < 0.05, uncorrected for multiple comparisons. There were no significant correlations at a corrected *p*-value of 0.002. The results are displayed in **Table 3**. Significant correlations for the double-marked test items and *left* BA44 occurred in scans 2–4 (see **Table 3**). These also occurred for single-marked words in Scans 2 and 4. Test performance was correlated with the *right* BA44 in Scan 2 for double-marked and Scans 1 for single-marked activations. In contrast, test performance was correlated for *right* BA45 during Scan 4, in IC 5 only. Additional correlations (*p* < 0.05 uncorrected) were found for 10 other consistently activated regions (80 total correlations) as well, but no one region correlated consistently with performance across scans. Instead, regions that predicted performance shifted over the learning period. None of these survived alpha correction to *p* < 0.0006.

**Table 3:** Brain-behavior correlations behavioral performance and independent components (IC 1–5).

		Double marked words				Single marked words			
Region	Hemisphere	Scan 1	Scan 2	Scan 3	Scan 4	Scan 1	Scan 2	Scan 3	Scan 4
Angular Gyrus	Left		IC 3: -0.54				IC 1: -0.49		
BA44	Left		IC 3: -0.68		IC 4: -0.53		IC 4: -0.51	IC 2: 0.48	
BA44	Right		IC 3: -0.71			IC 4: 0.51			
BA45	Right				IC5: -0.50				
Crus II	Left		IC 2: -0.50			IC 3: 51			
Crus II	Right	IC 1: 0.49	IC 2: -0.50						
Insula	Left	IC 3: 0.60							
Insula	Right		IC 4: -0.49		IC1: -0.65				
MFG	Right						IC 4: -0.50		IC1: -51
MTG-posterior	Right		IC 4: -0.50						
MTG-temp/occip	Left				IC 1: -0.67	IC 4: -0.54	IC 1: 0.59		IC 2: -0.67
MTG-temp/occip	Right			IC 2: -0.52					IC 4: 0.48
STG-posterior	Right	IC 1: -0.50	IC 2: 0.61				IC 2: 0.55	IC 1: 0.53	

*BA44: Inferior Frontal Gyrus-Pars Opercularis. BA45: Inferior Frontal Gyrus-Pars Triangularis. MFG: Middle Frontal Gyrus. MTG-temp/occip: Middle Temporal Gyrus-temporal-occipital region. STG-posterior: Posterior Superior Temporal Gyrus. All correlations are significant at *p* < 0.05, uncorrected for multiple comparisons.*

## Discussion

### Behavioral Learning

The behavioral results indicated that participants were able to respond to test items accurately only after a brief exposure to examples of Russian root words and their gender-marked suffixes. Moreover, learning reflected generalization of the patterns to untrained words. However, there was a noteworthy dissociation between learning of double- and single-marked patterns. Morphological patterns for double marked words were easier to generalize overall than the patterns for single marked words. This has also been reported by previous studies that have used similar Russian stimuli (Gerken et al., 2005; Richardson et al., 2006; Eidsvåg et al., 2015). Learning the double-marked pattern only required learning the association between the *–tel* and *–k* markers and the two suffixes each are paired with (i.e., tel+ya/yme; k+u/oj). Single-marked patterns required noting that root words that took –ya could also take –yem but those that took –u also took –oj. Thus, it is the relation between the single marked suffixes through their occurrence on many different root words that is required for single-marked category learning, making it a more difficult dependency to track than the adjacent dependency needed to learn the double-marked words.

Behavioral performance for Scan 1 indicated participants were tracking information relevant to both single and double-marked words simultaneously. This is consistent with the idea that multiple distributional cues in the input can be tracked simultaneously (De Diego Balaguer et al., 2007; Mueller et al., 2009; Havas et al., 2017). However, the drop in performance on single-marked nouns after Scan 1 suggests that learners developed an implicit strategy that resulted in increased learning of the easier of the two distributional relationships after the initial exposure. An implicit focus on easier patterns (i.e., double-marked words) may facilitate later learning of more difficult patterns (i.e., single-marked words). This is consistent with behavioral data in which learners bootstrapped learning of difficult patterns on successful learning of related but easier patterns (Lany et al., 2007). When input contains multiple dependencies, as was the case here, there is both behavioral evidence (Romberg and Saffran, 2013) and physiological evidence (De Diego Balaguer et al., 2007) that learning of the easier of the dependencies emerges first. Moreover, when there is more than one type of statistical dependency is present in the input, brain-behavior relations also shift over time (De Diego Balaguer et al., 2007; Plante et al., 2014).

When brain-behavior relations are considered (**Table 3**), there are relatively few correlations between activation and performance for the first scan. The behavioral results after Scan 1 indicate that the learners as a group showed above chance performance, despite chance pretest performance levels. Presumably, therefore, their brains assembled networks that were capable of learning. However, the learning network was likely still emerging. Electrophysiologic evidence suggests naïve learners acquiring grammatical information in a statistical learning format can achieve high behavioral accuracy while still showing physiologic responses that differ from native language users (Mueller et al., 2009; Foucart and Frenck-Mestre, 2012). Consistent with the idea that the early neural network is not yet stable, transitional physiological responses have been reported in a study that involved gender class learning (Foucart and Frenck-Mestre, 2012). We propose that the dynamic shifts in level and location of activation in the learning network, as well as correlations with behavior, represent physiologic attempts (successful or otherwise) to optimize the learning network. This physiologic reconfiguration during early learning likely contributed to the non-linear growth in behavioral performance at the group level, as well as shifts in brain-behavior correlations.

The relation between activation and performance during learning is known to be variable and complex. Learning has been associated with dynamic changes in regional activation over time (Plante et al., 2014, 2015, 2017). This is often not seen because many fMRI studies use a single learning period rather than track activation over time. Activation that predicts learning can also change over the initial exposure period (Plante et al., 2014). The nature of these brain-behavior correlations is not straight-forward. Increased activation with increased performance has been reported for word segmentation studies (McNealy et al., 2006, 2010; López-Barroso et al., 2015) and learning grammatical relationships (De Diego Balaguer et al., 2007). However, this positive relation between activation and performance is not universal. For example, higher than typical activations have been associated with poorer performance for participants with poor language or learning skills (Plante et al., 2006, 2014, 2017; Hoeft et al., 2007). This suggests compensatory over-activation occurs when learning is particularly difficult for the groups of learners (Hoeft et al., 2007). Habituation is also associated with lower activation over time as the brain requires less activation to process increasingly familiar stimuli (e.g., Celsis et al., 1999; Joanisse et al., 2007; Plichta et al., 2012). Habituation-like physiologic changes are seen even within the first minutes of language learning tasks (e.g., De Diego Balaguer et al., 2007; Cunillera et al., 2009) as learners succeed at the task. Cunillera et al. (2009) proposed an inverted U shaped physiologic function to describe an initial increase in activation as learning initiates and a decrease as stimuli become recognized as units. Mathematically, at least, increasing and decreasing levels of activation for different learners within a group could average out, yielding little or no correlation in a region that is actually directly contributing to behavioral performance. This would explain why, as reported here, significant correlations are found during some learning periods but not others in studies that tracked over time (Plante et al., 2014) or across samples (López-Barroso et al., 2015). Therefore, neither positive nor negative correlations are necessarily consistent with better learning, and lack of a correlation does not necessarily mean that a region was not contributing to learning.

In the present study, all the ICs represented task-dependent signal, in that greater activation occurred within the learning blocks compared with the control blocks. In this broad sense, all ICs were associated with periods of learning. For individual brain regions within each IC, both negative and positive correlations with behavior occurred. The strength and even direction of these correlations shifted over time. This may reflect that the region’s relative contribution was changing with time, that learners differed over time in how they were leveraging regions within the network over time, or both. We also saw that some right hemisphere activations correlated with behavior. Right hemisphere activation can occur because increased difficulty results in greater recruitment of right hemisphere in addition to the left. Behavioral correlations with the right hemisphere may reflect the relative recruitment of the non-dominant hemisphere, driven by task difficulty, rather than a unique contribution of this hemisphere itself. The relative difficulty of the learning task for the learner over time, the amount of effort required of neural systems to meet task difficulty, and the general evolution toward a stable network state over time may all lead to relative changes in brain-behavior relations across time and across brain regions.

### Network Activation

Activation during the statistical learning process was organized into five sub-networks each of which showed statistically independent BOLD signal time courses (i.e., ICs). For each of these, the BOLD signal cycled with the experimental stimulus blocks (Russian words vs. Tones). These task-related networks included regions that were differentially distributed across cortical, subcortical, and cerebellar regions. However, a small number of regions were common to multiple sub-networks, most notably the inferior frontal gyrus-pars opercularis, the middle frontal gyrus, and regions comprising the temporal-parietal-occipital junction. These regions were consistently active across ICs in Scans 1–4, suggesting they are central to the network. These findings correspond to those of Tettamanti et al. (2002), who also identified the pars opercularis, precentral, and angular/surpramarginal regions for their grammar learning task. These general similarities between these two studies occurred despite differences in language modality (auditory vs. visual) and the presence or absence of ungrammatical exemplars during the learning period.

This result supports our prediction that this region would be critical to learning a morpheme system in ways that were not consistently reported for statistical learning of uninflected word units segmented from continuous speech (McNealy et al., 2006, 2010; Karuza et al., 2013; López-Barroso et al., 2015; Plante et al., 2015). The present results can be compared directly with the results of our previous word segmentation study (Plante et al., 2015), in that the procedures for scanning and ICA analyses were identical. In that word segmentation study, activation in the pars opercularis was reported in the left hemisphere for two ICs and in the right hemisphere for four ICs. In each of these ICs, this region was only intermittently activated during periods of exposure to the input. Likewise, López-Barroso et al. (2015) reported inconsistent activation of inferior frontal gyrus in their word segmentation task. However, inconsistent activation may reflect the difference between requisite activation of regions, given the nature of the input, and transient activations that occur as the brain struggles to hone in on the optimal learning network.

In the current study, the consistent activation of the pars opercularis, across scans and in multiple ICs, suggests that it was acting as a hub for this learning task. Logically, within a neural network, there should be a means to pass information among sub-networks to produce coordinated functioning at the overall network level. The left pars opercularis, active in four out of the five ICs (ICs 1, 2, 4, and 5), is well positioned for this role. Other areas also showed overlap among ICs. The second most frequently occurring regions were in the –temporo-parietal-occipital junction bilaterally, which showed overlap in two ICs each (ICs 1 and 3). As with the inferior frontal regions, these areas were also consistently active in all four scans during the learning period, suggesting these regions were also central to the learning task. These regions have been reported for other statistical learning paradigms (e.g., Plante et al., 2014, 2015, 2017; López-Barroso et al., 2015), indicating they have a general, rather than input-specific role in this type of learning.

If the inferior frontal gyrus plays a role specifically in implicit learning of inflectional “rules,” then the posterior regions are likely to support the phonological processing that is pre-requisite to morphological processing. The supramarginal and angular gyri appear to contribute differentially to auditory processing. Activation in the supramarginal gyrus modulates depending on the listener’s level of attention during word processing (Christensen et al., 2012), which is consistent with the idea that posterior temporal-parietal regions are critical for placing phonological information within the focus of attention (Chien et al., 2003). The angular gyrus shows greater activation to new auditory words compared to words heard before (Christensen et al., 2012). Activation that includes both the supramarginal and angular gyri is higher for pseudo-words than for real words (Clark and Wagner, 2003) as well as backward speech than for forward speech (Karuza et al., 2013). These regions are also both active when acoustic changes signal different phonological categories (Turkeltaub and Coslett, 2010). In all these cases, activation in these areas appears to rise as the signal diverges from familiar or prototypical phonological material and more listener resources are required. This function would be critical in a learning context such as ours as listeners encounter accented speech, foreign words, and a phonology-based morphological system for marking gender. Although activation in this area has not been reported during word segmentation tasks analyzed with GLM analysis (McNealy et al., 2006, 2010, 2011; Karuza et al., 2013), both ICA studies (López-Barroso et al., 2015; Plante et al., 2015) reported supramarginal and angular gyrus activation in multiple ICs. Therefore, the lack of activation in previous GLM studies may reflect lack of analytic power rather than a lack of engagement of these regions.

We suggest that the core statistical learning network for morphological patterns as seen here is actually the same as the network that is ultimately activated when skills have been acquired. This is consistent with the theoretical position that statistical learning is the mechanism by which language is established as a cognitive system. However, the present results, when compared with previous studies of statistical learning, strongly suggest that there is not one statistical learning module or network. Instead, the statistical learning network appears to organize around the input it receives. The present study demonstrated robust recruitment of the inferior frontal gyrus that was not present in previous studies of word segmentation from running speech (McNealy et al., 2006, 2010, 2011; Karuza et al., 2013; Plante et al., 2015). In the present study, participants were given single words during the input and during the test phases, but were not made aware that the target of learning was a morphological pattern that cued word subclasses. Yet, from the first scan on, the inferior frontal gyrus was activated for this input. This strongly suggests that networks leveraged for statistical learning are driven, from the start, by the nature of the input rather than strategies that participants develop to drive their learning. This builds on the finding of Plante et al. (2015) who reported highly distinct networks for input that either facilitated or restricted the possibility of statistical learning. Input that allowed for statistical learning engaged a much more widely distributed network compared to nearly identical input from which key input statistics were not present. This occurred even though the participants were blind to the nature of the input. The results of the present study indicate that even when statistical information is present, the network assembled is driven by fairly specific aspects of the input.

The inferior frontal gyrus in particular appears to be differentially engaged in statistical learning, depending on the nature of the pattern to be learned. Here, we have contrasted morpheme learning with the segmentation of words in running speech. The inferior frontal gyrus appears to be preferentially engaged in learning phonological patterns that signal morphological information. For this inflection pattern, certain gendered morphemes predicted the immediate occurrence of a specific subset of additional gender morphemes. This type of co-occurrence is considered an adjacent contingency in statistical learning parlance. Activation of the inferior frontal gyrus has also been reported for implicit syntactic learning (Tettamanti et al., 2002), and with auditory, but non-verbal stimuli (Barascud et al., 2016). In each of these studies, the dependencies represented in the input were among adjacent elements in a sequence. These common findings across similar input dependencies suggest that bound morpheme learning may require application of a more general statistical learning mechanism, rather than different mechanisms for linguistic and non-verbal learning. Furthermore, the role of the inferior frontal gyrus’ may be that of “a generic on-line structured sequence processor that unifies information from various sources…” (Petersson et al., 2012, p. 89). The stimuli used in the present study required unification of information across elements presented in the input (e.g., the suffix -tel will also take either–ya or –yem, but not –oj or –u). This type of learning can be differentiated from the type of sequential pattern learning attributed to activation of the superior temporal gyrus in word segmentation studies (Plante et al., 2015; Barascud et al., 2016). In word-segmentation paradigms, sequenced elements consisted of syllable dependencies, such that syllables composing one word were never encountered within another word. Therefore, every “word” is phonologically unique in relation to every other word in the input. In contrast, patterns defining both syntactic and morphological patterns are distributional in nature. The evidence for distributional patterns requires calculation of related, but not always identical contingencies over a set of informative input.

It remains to be seen if other morphological arrangements show a similar pattern of frontal activation. For example, some morphological markings reflect non-adjacent contingencies, as in subject-predicate agreement marking in English. Free morphemes, such as articles and auxiliaries, may require a different network than that described for Russian gender marking. Nevat et al. (2017) reported findings for the inferior frontal gyrus that may indicate this type of regional variation is possible. Nevat et al. (2017) reported significant activation during the test phase of a study in which participants were previously trained on an artificial grammatical marking system using an exposure-practice-feedback format. They reported non-significant activation in pars opercularis, with significant activation appearing in pars triangularis. Although there are important methodological differences between their study and the present work, the findings of Nevat et al. (2017) raise the possibility that subtle differences in regional activation may correspond to the distributional properties of the morphology to be learned.

This basic statistical language-learning system are accompanied by activations in areas generally associated with memory and attention (e.g., cingulate cortex, dorsolateral prefrontal cortex). Again, these activations are more commonly seen in ICA studies (López-Barroso et al., 2015; Plante et al., 2015), including the present study, than in those that have used GLM analyses. However, these additional activations are consistent with the tenets of the statistical learning framework, in that attention to and memory for input characteristics is necessary for this type of learning (Erickson and Theissen, 2015). The ICA studies have also indicated that a number of transient activations occur during learning. Some have suggested that early activation that disappears is involved only during initial learning (Nevat et al., 2017). However, transient activations also occurred after the initial learning period here and elsewhere for implicit language learning tasks (Plante et al., 2014, 2015; López-Barroso et al., 2015). It is unknown the extent to which these transient activations actually contribute to the formation of an effective learning network. If certain transient activations relate to a particular learning stage, they should replicate across studies. Activations that fail to replicate are likely to reflect random changes within the brain as it attempts to form stable networks that are effective and efficient for the learning task.

## Ethics Statement

The work was reviewed by the University of Arizona Institutional Review Board. Adults received oral and written information concerning the study and indicated their willingness to participate by signing approved consent documents.

## Author Contributions

MS contributed to the data collection and analysis and was the lead in manuscript preparation. EP designed the study and contributed the next most to the manuscript writing. DP contributed to the analysis and results. HD contributed to the statistical analysis and conceptualization of the results. CV contributed to the analysis and figure preparations. All authors contributed to the text

## Conflict of Interest Statement

The authors declare that the research was conducted in the absence of any commercial or financial relationships that could be construed as a potential conflict of interest.
