# Supplementary material for: Neural Correlates of Morphology Acquisition through a Statistical Learning Paradigm
Source: Front Psychol. 2017 Jul 27;8:1234. doi: 10.3389/fpsyg.2017.01234 (PMC5529410; doi:10.3389/fpsyg.2017.01234)
Supplement: Supplementary file 1 [file Table_1.PDF]

Supplemental Table 1. Regions of significant activation by IC and scan number. Scans were obtained sequentially within the same session. Regions are those defined by the Harvard-Oxford cortical and subcortical structural atlases and the Probabalistic Atlas of the Human Cerebellum. Activation within each of these pre-defined regions was tested for significance at $p<.05$. This ROI-based analysis provides a similar result as brain-wide correction for multiple comparisons (compare with Figure 2). Regions without values for a particular scan were those without significant activation during that scan. Coordinates (X, Y, Z) refer to MNI space, given 2x2x2mm voxels.

				**Scan 1**				**Scan 2**				**Scan 3**				Scan 4			
**IC**	**Hemis.**		**Region**	**Peak t**	**X**	**Y**	**Z**	**Peak t**	**X**	**Y**	**Z**	**Peak t**	**X**	**Y**	**Z**	**Peak t**	**X**	**Y**	**Z**
1	L	Frontal	Frontal Medial Cortex	8.16	-42	12	24	10.23	-52	20	12	8.06	-52	20	12	8.34	-48	14	0
			Frontal Opercular Cortex	9.67	-50	24	14	9.90	-52	22	12	8.51	-46	30	-4	7.75	-50	24	-4
			Frontal Orbital Cortex													3.81	-2	36	-10
			Inferior Frontal Gyrus: pars opercularis	9.84	-38	26	0	11.35	-46	22	-4	16.61	-42	24	-2	11.10	-46	24	-4
			Inferior Frontal Gyrus: pars triangularis	9.59	-38	24	-2	12.64	-46	22	-6	12.92	-42	24	-4	17.92	-48	20	-8
		Temporal	Angular Gyrus	14.96	-50	-52	18	13.78	-60	-50	14	14.74	-62	-50	12	15.02	-54	-56	14
			Inferior Temporal Gyrus: temporo-occipital	5.03	-44	-50	-8	5.08	-44	-52	-16					2.51	-42	-16	-22
			Middle Temporal Gyrus: anterior	6.71	-56	-8	-12	8.17	-52	2	-20	8.10	-58	-2	-14	11.72	-60	-8	-12
			Middle Temporal Gyrus: posterior	10.98	-60	-24	-6	12.90	-54	-26	-6	12.79	-54	-26	-6	11.29	-50	-24	-8
			Middle Temporal Gyrus: temporo-occipital	18.94	-56	-46	8	14.59	-60	-48	8	16.79	-60	-50	10	17.95	-58	-46	8
			Planum Polare									7.17	-50	4	-6				
			Planum Temporale	12.04	-60	-36	14	11.03	-60	-32	10	11.44	-52	-30	6	12.64	-56	-32	8
			Superior Temporal Gyrus: anterior	12.26	-54	-8	-6	13.32	-56	-8	-2	12.76	-56	-2	-10	10.81	-60	-10	-4
			Superior Temporal Gyrus: posterior	15.55	-58	-20	-4	17.57	-54	-24	-2	14.78	-60	-24	0	15.18	-48	-24	-6
			Temporal Pole	9.97	-48	16	-14	10.44	-50	18	-12	13.15	-54	8	-14	10.76	-48	18	-12
		Parietal	Precuneous Cortex	8.57	-2	-58	54					10.69	-4	-48	52				
			Parietal Opercular Cortex	7.25	-60	-40	26	6.36	-60	-40	26	5.85	-60	-40	24	7.61	-60	-40	26
			Supramarginal Gyrus: posterior	22.47	-56	-46	10	14.94	-62	-48	10	22.04	-58	-48	12	16.36	-60	-46	8

		Cerebellum	Crus II													9.21	-10	-78	-48
			VIIb									10.17	-22	-78	-40	10.07	-18	-82	-34
	R	Frontal	Frontal Opercular Cortex	10.96	50	26	-6	16.85	48	22	-6	9.19	42	20	-6	8.10	36	32	-2
			Frontal Orbital Cortex	8.38	50	22	16	8.41	50	20	28	9.72	48	12	28	8.32	50	16	2
			Inferior Frontal Gyrus: pars opercularis	11.55	52	22	-6	12.57	50	26	-2	10.00	46	30	4	14.21	52	34	2
			Inferior Frontal Gyrus: pars triangularis	9.69	48	22	-4	17.46	46	26	-2	9.65	46	26	2	9.76	48	26	0
			Middle Frontal Gyrus	7.77	44	4	46	8.91	44	16	34	8.89	38	2	54	9.09	48	8	40
		Temporal	Angular Gyrus	16.97	52	-54	14	18.11	56	-44	22	18.42	58	-46	22	18.70	50	-44	22
			Inferior Temporal Gyrus: temporo-occipital									5.00	46	-44	-12				
			Middle Temporal Gyrus: anterior	9.55	58	-6	-14	13.91	56	-2	-18	8.94	58	-6	-14	10.33	56	-4	-16
			Middle Temporal Gyrus: posterior	18.50	56	-12	-12	19.82	50	-30	-4	21.65	50	-30	-6	20.37	50	-12	-12
			Middle Temporal Gyrus: temporo-occipital	14.17	50	-38	4	14.35	52	-40	4	14.70	52	-40	4	12.98	56	-40	4
			Planum Polare					12.22	50	-10	-6								
			Planum Temporale	12.68	52	-24	6	11.31	52	-32	12	14.15	56	-36	18	11.26	56	-36	18
			Superior Temporal Gyrus: anterior	14.76	56	-6	-6	20.94	56	0	-14	16.50	50	-6	-10	13.94	50	-2	-12
			Superior Temporal Gyrus: posterior	22.58	58	-10	-8	23.54	48	-24	-2	20.55	50	-10	-10	20.88	56	-10	-10
			Temporal Pole	11.94	50	6	-16	11.40	46	18	-24	11.66	52	10	-18	11.83	50	10	-22
		Parietal	Precuneous Cortex	8.60	2	-62	48	8.82	2	-54	50	9.33	4	-60	50	9.05	2	-54	56
			Supramarginal Gyrus: posterior	13.27	58	-44	18	15.57	50	-40	6	22.40	50	-42	16	16.20	58	-44	22
		Subcortical	Thalamus	8.59	70	-30	4												
2	L	Frontal	Frontal Medial Cortex	7.40	-2	36	-14	6.88	-2	36	-8	7.99	-2	48	-10	7.68	-4	48	-10
			Inferior Frontal Gyrus: pars opercularis	11.36	-56	18	28	8.01	-56	18	28	10.99	-56	16	26	9.08	-46	18	18
			Inferior Frontal Gyrus: pars triangularis	11.79	-48	30	4	10.58	-54	28	12	11.32	-48	30	14	9.69	-46	30	8

		Temporal	Inferior Temporal Gyrus: posterior					3.37	-44	-20	-20								
			Inferior Temporal Gyrus: temporo-occipital	6.19	-46	-62	-12	6.79	-46	-58	-16	8.95	-50	33	-14	6.13	-56	-50	-12
			Temporal Occipital Fusiform Cortex					6.63	-44	-60	-14					5.94	-28	-48	-12
		Cerebellum	Vermis Crus II	4.63	-2	-78	-28	3.31	-2	-78	-28	3.20	-2	-78	-30				
			Vermis IX									3.50	-2	-56	17				
			Vermis VIIb									2.92	-4	-68	-32				
			Vermis X									3.09	-2	-48	17	3.05	-2	-46	-36
	R	Frontal	Frontal Medial Cortex	7.612443	8	52	-10	7.81	4	54	-10	8.47	8	52	-8	6.73	6	48	-14
			Inferior Frontal Gyrus: pars opercularis	17.719755	52	20	28	13.75	50	14	32	14.47	52	14	30	17.04	52	22	26
			Inferior Frontal Gyrus: pars triangularis	10.271517	48	30	20	9.61	50	30	24	10.69	48	30	20	17.73	50	30	22
			Middle Frontal Gyrus													28.05	50	28	26
		Temporal	Inferior Temporal Gyrus: temporo-occipital	6.059505	50	-46	-12	4.62	50	-48	-12	4.65	50	-50	-20				
			Temporal Fusiform Cortex anterior					3.28	36	-6	-32								
		Cerebellum	Vermis IX	5.822894	2	-48	-40					4.24	2	-48	17				
			Vermis X									4.61	2	-44	-38	2.77	2	-46	-38
			X					4.64	16	-36	-50								
3	L	Frontal	Middle Frontal Gyrus	9.42	-42	28	42	13.70	-30	14	56	9.46	-32	24	46	15.92	-36	24	46
			Superior Frontal Gyrus					14.59	-2	34	44					10.60	-2	30	52
		Temporal	Angular Gyrus	11.59	-56	-52	44	10.58	-56	-60	34	11.26	-50	-52	54	11.35	-56	-60	36
			Heschl's Gyrus (includes H1 and H2)									5.69	-48	-22	8	9.41	-48	-18	8
		Parietal	Supramarginal Gyrus: posterior	11.01	-54	-50	46	12.31	-54	-48	48	10.66	-56	-46	44	12.85	-48	-46	48
		Cerebellum	Crus I	14.30	-12	-82	-22	14.86	-32	-66	-30	13.89	-8	-80	-24	11.78	-34	-70	17
			Crus II	11.79	-36	-72	-46	19.32	-38	-64	-44	13.69	-10	-80	-32	15.59	-34	-68	-42

			IX	6.79	-4	-58	-56					10.12	-6	-56	-54				
			Vermis VI													5.76	-4	-78	-20
			Vermis IX									3.63	-2	-56	-32				
			Vermis X	4.64	-2	-46	17	4.11	-2	-48	-32	3.47	-2	-48	-34	4.19	-2	-46	17
	R	Frontal	Frontal Opercular Cortex	9.12	46	20	-4	9.68	48	20	0	10.17	44	22	-2	11.38	42	22	-2
			Frontal Orbital Cortex	11.36	38	22	-6	7.85	30	22	-8	10.80	38	26	-6	12.07	42	22	-4
			Frontal Pole	13.45	8	38	54	17.33	46	56	4	15.88	32	58	6	18.58	42	52	-12
			Inferior Frontal Gyrus: pars opercularis	9.27	52	16	32	10.24	50	14	32	10.71	50	14	32	9.14	52	14	32
			Inferior Frontal Gyrus: pars triangularis	7.39	50	28	18	9.22	50	30	20	9.25	52	28	16	7.97	50	20	-2
			Middle Frontal Gyrus	16.71	28	20	56	19.97	48	34	34	18.29	48	32	36	23.06	48	26	38
			Paracingulate Gyrus	15.40	4	36	38	16.30	6	38	38	17.66	2	34	42	14.00	2	40	36
			Superior Frontal Gyrus	18.43	4	34	50	18.07	8	36	42	21.35	34	26	52	20.13	20	26	50
		Parietal	Cingulate Gyrus: posterior	15.46	6	-44	36	10.88	2	-28	40	12.86	4	-42	36	12.70	2	-26	40
		Temporal	Angular Gyrus	19.78	50	-50	42	22.76	50	-50	42	19.19	50	-48	34	17.15	50	-46	48
			Inferior Temporal Gyrus: temporo-occipital									4.96	52	-44	-12	6.68	50	-50	-18
			Middle Temporal Gyrus: posterior	8.12	66	-24	-6	9.43	64	-24	-8	11.78	58	-24	-6	10.49	66	-26	-4
			Middle Temporal Gyrus: temporo-occipital	6.86	62	-50	12	6.43	64	-40	-2	8.03	64	-40	-2	8.16	60	-42	0
			Precuneous Cortex	12.13	8	-68	44	12.99	8	-56	42	10.86	4	-50	34	10.30	4	-54	40
			Supramarginal Gyrus: posterior	16.25	50	-44	50	20.38	50	-44	46	16.09	50	-44	44	19.01	50	-44	48
		Occipital	Lateral Occipital Cortex superior	17.63	50	-60	44	15.85	50	-62	40	16.72	50	-58	42	13.79	42	-56	56
		Subcortical	Caudate	8.52	39	8	20	6.70	39	10	18	9.70	39	10	14	9.25	39	10	12
			Thalamus					8.16	4	-6	14								
		Cerebellum	Vermis X	3.78	2	-44	17	4.31	2	-46	-36								
4	L	Frontal	Inferior Frontal Gyrus: pars opercularis	10.22	-50	12	0	9.54	-50	10	2	7.96	-48	10	4	8.57	-50	10	4

		Central Opercular Cortex	11.11	-38	67	6												
		Cingulate Gyrus: anterior	14.64	-2	28	30	16.38	-2	16	36	19.27	-4	20	34	15.13	-4	16	38
		Frontal Opercular Cortex	9.70	63	18	8	15.12	-36	16	6	10.85	-36	22	2	10.46	-34	18	6
		Frontal Pole	14.49	46	44	30					13.37	-32	48	30				
		Juxtapositional lobule Cortex*	15.67	-4	8	52	19.22	-2	8	56	17.40	-4	2	66	21.01	-2	4	64
		Middle Frontal Gyrus	12.10	46	16	54	14.25	44	34	38	17.16	-32	36	38	15.74	-34	14	52
		Paracingulate Gyrus	18.82	-2	30	38	16.90	-4	18	38	21.14	-6	22	38	17.27	-2	18	50
		Precentral Gyrus	11.86	-42	-2	48	11.77	-30	-8	62	13.58	-42	-8	50	13.71	-44	-14	54
		Superior Frontal Gyrus	16.94	-2	26	46	13.76	-2	10	56	16.80	42	16	58	18.60	-2	18	52
	Insular	Insular Cortex	10.59	63	67	6	13.31	-32	16	4								
	Temporal	Inferior Temporal Gyrus: posterior					3.08	-44	-28	-18	3.10	-44	-24	-20				
		Inferior Temporal Gyrus: temporo-occipital	6.02	-50	-52	-16												
		Temporal Occipital Fusiform Cortex													4.30	-34	-56	-20
	Occipital	Intracalcarine Cortex									5.70	-4	-72	10	4.93	-8	-84	8
	Cerebellum	Vermis VI									6.36	-2	-70	-14	3.65	-4	-68	-18
		Vermis X	3.80	-2	-44	17	3.77	-2	-46	17	4.32	-2	-44	17				
R	Frontal	Central Opercular Cortex					12.22	44	8	6	8.27	48	8	4				
		Cingulate Gyrus: anterior	12.14	6	18	36	16.14	2	14	38	15.83	4	28	32	13.96	6	16	38
		Frontal Medial Cortex					4.31	8	42	-16								
		Frontal Opercular Cortex	10.24	42	12	2	10.15	44	10	6	10.19	38	18	4	11.87	38	16	6
		Inferior Frontal Gyrus: pars opercularis					10.44	50	14	2	8.00	52	10	4	7.84	50	10	2
		Juxtapositional lobule Cortex*	16.78	6	8	56	17.14	2	8	56	19.19	4	4	60	21.98	2	2	60
		Middle Frontal Gyrus	14.58	28	6	58	11.46	26	30	44	14.59	36	0	65	16.08	27	0	56
		Precentral Gyrus	10.80	50	2	46	13.57	46	2	46	15.12	50	-8	44	18.51	36	-2	46
		Superior Frontal Gyrus	20.53	4	10	56	14.29	2	10	65	15.93	2	14	54	24.32	2	16	54
		Paracingulate Gyrus	17.41	2	30	38	17.32	4	14	46	17.16	2	12	52	19.83	8	18	50
	Temporal	Inferior Temporal Gyrus:					2.42	44	-28	-18								

			posterior																
		Parietal	Supramarginal Gyrus: posterior					6.42	64	-38	26								
		Subcortical	Globus Pallidus-Central													5.02	24	-2	-4
		Cerebellar	Vermis VI													4.69	2	-72	-12
			Vermis X	3.75	2	-44	17												
			VIIb									4.81	26	-68	-54				
5	L	Frontal	Central Opercular Cortex	10.91	-38	-10	14	9.56	-56	-12	14	10.91	-60	-12	14	9.90	65	-12	14
			Inferior Frontal Gyrus: pars opercularis					7.13	-50	10	24	7.40	-48	10	28	7.97	-54	12	24
			Precentral Gyrus	12.53	71	-10	48	11.01	-54	48	22	14.53	-58	2	24	14.69	-48	48	38
			Subcallosal Cortex	5.57	-2	26	-6												
		Temporal	Temporal Occipital Fusiform Cortex																
		Parietal	Postcentral Gyrus	12.27	-54	-12	36	11.24	-58	-10	24	13.21	-60	-12	30	21.89	-56	-14	38
		Cerebellum	Vermis X									2.30	-2	-46	17				
	R	Frontal	Central Opercular Cortex	11.52	44	-8	14	8.62	38	-6	16	12.07	38	-8	18	12.06	40	-6	16
		Parietal	Postcentral Gyrus					9.77	60	-10	44								

*formerly Supplementary Motor Cortex
