# Supplementary material for: Neural Correlates of Morphology Acquisition through a Statistical Learning Paradigm
Source: Front Psychol. 2017 Jul 27;8:1234. doi: 10.3389/fpsyg.2017.01234 (PMC5529410; doi:10.3389/fpsyg.2017.01234)

Supplemental Figure 1. fMRI data (t-values) analyzed using a GLM approach. Image data was obtained on a 3T Siemans Skyra with a 32-channel head coil using the acquisition and the preprocessing methods specified in the methods of this paper. GLM processing used AFNI (ordinary least squares regression) for language and tone blocks. The AFNI default BOLD model as the ideal waveform. Beta coefficients for each voxel were tested for significance using voxel-wise t-statistics. Images were thresholded at activation levels and activation cluster sizes that held FWE rates to p<.05 (corrected). The resulting image reflects t values with hotter colors reflecting higher values.

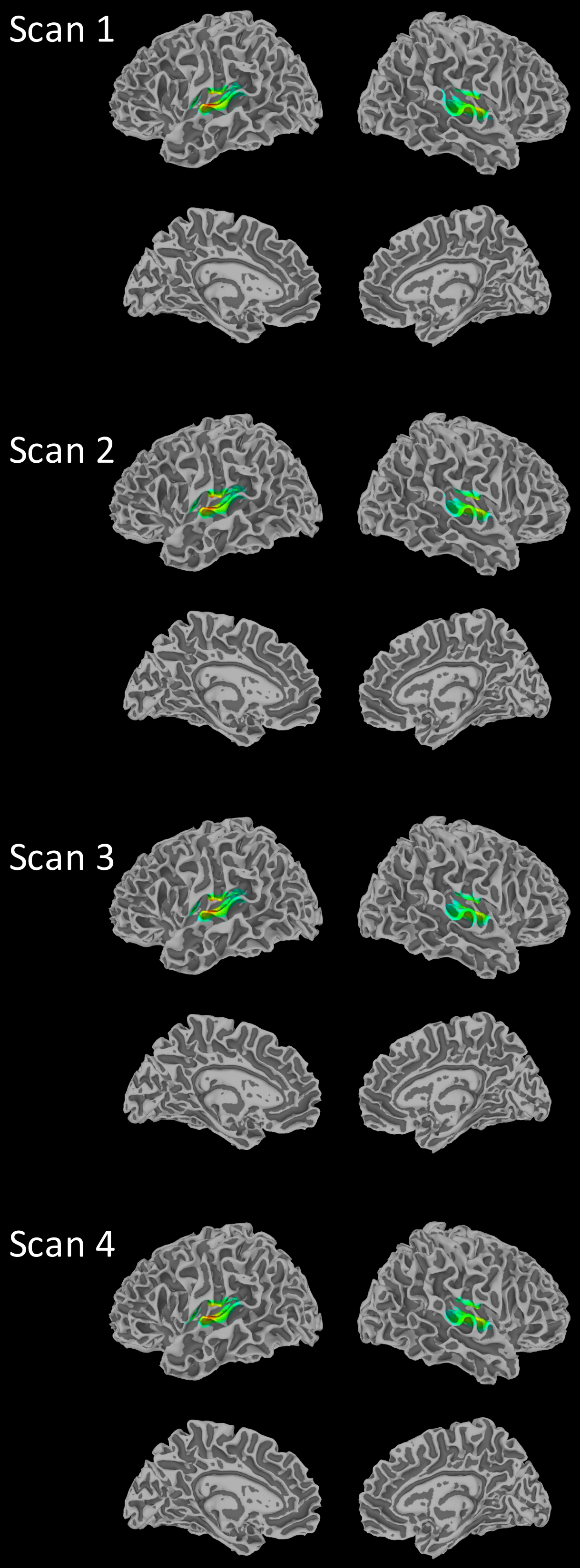

Supplement: Supplementary file 2 [file Image_1.PDF]
